# Mouse Models in Prostate Cancer Translational Research: From Xenograft to PDX

**DOI:** 10.1155/2016/9750795

**Published:** 2016-05-18

**Authors:** Domenica Rea, Vitale del Vecchio, Giuseppe Palma, Antonio Barbieri, Michela Falco, Antonio Luciano, Davide De Biase, Sisto Perdonà, Gaetano Facchini, Claudio Arra

**Affiliations:** ^1^Animal Facility Unit, Department of Experimental Oncology, Istituto Nazionale per lo Studio e la Cura dei Tumori “Fondazione G. Pascale”, IRCCS, 80131 Naples, Italy; ^2^Department of Veterinary Medicine and Animal Productions, Università di Napoli Federico II, Via Delpino 1, 80137 Naples, Italy; ^3^Department of Urology, Istituto Nazionale per lo Studio e la Cura dei Tumori “Fondazione G. Pascale”, IRCCS, 80131 Naples, Italy; ^4^Division of Medical Oncology, Department of Uro-Gynaecological Oncology, Istituto Nazionale per lo Studio e la Cura dei Tumori “Fondazione G. Pascale”, IRCCS, 80131 Naples, Italy

## Abstract

Despite the advancement of clinical and preclinical research on PCa, which resulted in the last five years in a decrement of disease incidence by 3-4%, it remains the most frequent cancer in men and the second for mortality rate. Based on this evidence we present a brief dissertation on numerous preclinical models, comparing their advantages and disadvantages; among this we report the PDX mouse models that show greater fidelity to the disease, in terms of histopathologic features of implanted tumor, gene and miRNA expression, and metastatic pattern, well describing all tumor progression stages; this characteristic encourages the translation of preclinical results. These models become particularly useful in meeting the need of new treatments identification that eradicate PCa bone metastases growing, clarifying pathway of angiogenesis, identifying castration-resistant stem-like cells, and studying the antiandrogen therapies. Also of considerable interest are the studies of 3D cell cultures derived from PDX, which have the ability to maintain PDX cell viability with continued native androgen receptor expression, also showing a differential sensitivity to drugs. 3D PDX PCa may represent a diagnostic platform for the rapid assessment of drugs and push personalized medicine. Today the development of preclinical models in vitro and in vivo is necessary in order to obtain increasingly reliable answers before reaching phase III of the drug discovery.

## 1. Introduction

Despite the many scientific advances in pharmacological, clinical, and preclinical settings, prostate cancer (PCa) remains to be the first most common cancer in men [1]; it represents a major cause of cancer-related morbidity and mortality [2, 3]. In the United States there were estimated 233,000 (27%) new cancer prostate cases diagnosed in 2014, with an incidence of death valued at 29,480 (10%) patients annually [4]. Currently the radical prostatectomy is one of the gold standards for the treatment of PCa [5], despite the recent pharmacological approach with novel antineoplastic developed and approved drugs (enzalutamide and abiraterone) that target the androgen receptor axis [6, 7], and also immunologic therapies with antiangiogenic molecules used in patients with progression of disease [8]. The lethal cases generally have a high Gleason score and can be metastatic and/or refractory to androgen deprivation therapy (castration-resistant prostate cancer mCRPC); these have a short survival of 1–3 years, depending on context. Skeletal metastasis is the most significant cause of morbidity and mortality in PCa; it is found in approximately 90% of patients who die because of PCa [9]; this one indicates that the bone microenvironment may promote the growth of PCa cells. The majority of bone lesions in PCa usually show a greater proportion of active osteoblasts than active osteoclasts, resulting in the net formation of bone [10]. On the other hand, despite the many new therapies for patients with advanced CRPC, the overall survival is still relatively short [11] due to an endocrine therapy resistance development in a part of patients. These mechanisms include interference of androgen receptor (AR) axis and inhibition of androgens biosynthesis [1, 12, 13]. Malignant cells derived from prostate epithelial layers of PCa, which include secretory luminal, basal, and rare neuroendocrine cells, lend to disease a high grade of heterogeneity [9]. A question is pending about which type of epithelial cell represents the origin of PCa: luminal stem cells, basal stem cells, or both [14]; this point still remains unclear, but it is likely that there is a complex explanation of the heterogeneity of the disease and the many genetic pathways that are involved [15]. Despite the incidence rate trend in PCa, which continues to fall by 3-4% each year, and a large amount of prostate cancer studies, only very few findings have influenced the clinical management of the disease.

Innovative mouse models of prostate cancer have been developed to overcome the well-known limits and difficulties in PCa research [4]. For instance, the heterotopic models show some advantages like an easier tumor cells inoculation and in vivo tumor growth monitoring. While the heterotopic models mimic human prostate cancer in a more realistic way, the orthotopic implantation of tumor cells in the host more likely resembles the different tumor stages, dealing with native environment of tumor cells [16]. Independently from the advantages of these models in preclinical research, most studies are additionally hampered by a lack of standardization. Especially in orthotopic tumor cell application, the quality and number of inoculated tumor cells, as well as the addition of extracellular factors, have never been investigated in detail.

A major limitation in prostate cancer research is the lack of relevant preclinical models, which allow studying the molecular mechanisms of tumorigenesis. In fact, advanced in vitro and in vivo models are an indispensable requirement for the development of effective prevention and therapeutic intervention strategies [3]. To address and overcome the limitations of traditional models, reaching a greater loyalty compared to PCa human, currently patient-derived models (PDX) are used for preclinical research. Their use permits highlighting various aspects of PCa biology including angiogenesis, the identification of resistant castrate stem-like cells, and the effect of antiandrogen therapies [17]. PDX models are generated by using tumor tissues surgically removed from patient and plugged directly into the immune-compromised mice, without any manipulation in vitro. The tumors are subsequently maintained in vivo by mouse-to-mouse passages. Therefore we expect that PDX tumors models, which remain biologically stable and retain much of molecular, genetic, and histological characteristics, heterogeneity of original tumor, and response to treatment, become largely used in studies; instead, given the high costs of animal maintenance, lengthy latency period following engraftment, variable engraftment rates, and rare access to patient tissue specimens, PDX in vivo models are generally not yet widely employed in cancer research [18–20]. Mouse models can answer many questions about the etiology of cancer and help in screening new drugs and the development of more effective treatments. This work aims to collect and summarize data from research studies in order to identify, evaluate, and critically analyse in vivo models of PCa. Our ultimate goal was to obtain a systematic treatise about the progress as well as the advantages and disadvantages of these models in preclinical translational research.

## 2. Prostate Cancer Mouse Models

To date, preclinical prostate cancer mouse models still represent an essential tool to improve our understanding of PCa development, progression, and metastatic pattern. Spontaneous PCa transformation in different mouse strains is really uncommon [21]; therefore in the last years there have been created different generations of new manipulated mouse models of PCa to simulate all the stages of pathology, from the hyperplasia to HGPIN (high-grade prostatic intraepithelial neoplasia), until metastasis dissemination [21]; these transformation steps are slightly different from a histopathological point of view between human and mouse [22].

*Xenograft mouse models* (Table 1) represent a common “recipient” of human PCa, generated through orthotopic or heterotopic implantation of human tumor tissues, cell lines, or primary cell cultures, in nude mice [23], SCID [24], NOD-SCID [25], NOG/NSG [26], or RAG [27]. Different human prostate cancer cell lines have been used to perform various xenograft mouse models that, in this way, exhibit different features of the prostatic neoplasia. For instance LNCaP-LN3 cells, derived from androgen receptor mutated LNCaP [28], promote a high regional lymph node metastatic pattern after prostatic implantation, with castration-resistance features [29]. Instead PC3 cell lines, derived from bone metastasis of human PCa, are androgen-independent [30]; the PC-3M variant shows a higher ability to produce regional lymph node and distant organ metastasis [29].

*Allograft mouse models* (Table 1) represent a mouse tumor system that can be generated by using tumor cell lines/tissues derived from the same genetic background. In this way transplanted material and the host share the strain. TRAMP-C1/2/3 [31, 32] prostate cancer cell lines, derived from the prostatic epithelium of TRAMP mouse, and PTEN-CaP8 [33], from Pten-null transgenic mouse model of PCa, are both in strain C57BL/6; instead PNEC30, originated from neuroendocrine cell population in mouse prostate cancer, is in strain BALB/c [34].

*Genetically engineered mouse models (GEMMs)* (Table 2) reproduce in depth the different stages of PCa associated with typical human genetic mutation that allow tumor progression. We recognize two generations of PCa GEMMs, the first one is characterized by the ectopic expression of Simian virus 40 (SV40) Large Antigen T (Tag) in prostate; this effector acts as an oncoprotein that negatively regulates p53 [35, 36]. In 1994 the C3(1)-Tag model was generated, the first GEMM under the expression of SV40 large Tag; this model showed prostatic epithelial hyperplasia about 3 months of age and local adenocarcinoma by 7–11 months of age [37]. Also the TRAMP model (transgenic adenocarcinoma of the mouse prostate) is based on the SV40 Tag activity, under the prostate-specific rat Probasin promoter [38]; these mice develop epithelial hyperplasia, after 8 weeks of age, PIN (prostatic intraepithelial neoplasia) by 18 weeks, and lymph node metastasis by 28 weeks; this model has been the first that showed distant organ metastasis. The FG-Tag (fetal globin-*γ*/T-antigen) [39], LPB-Tag (LADY) [40], and LPB-Tag/ARR_2_PB-hepsin [41] chronologically represent the last example of first generation GEMM with a typical neuroendocrine cell transformation.

The second-generation GEMMs were born in 1997 with the Mt-PRL [42], a mouse model created to further study the role of prolactin (PRL) in prostate hyperplasia; the PRL ectopic expression has been inducted by Metallothionein-1 (Mt-1) promoter. Subsequently the BK5-IGF1 mouse models [43] were created which overexpressed the insulin-like growth factor 1, a molecule that resulted as an indicator of PCa in some patients that showed normal levels of PSA [62]. From 2001 there have been developed some other models using the Probasin (PB) promoter, among which are the following: PB-mAR [44], to further understand the role of androgen receptor on the disease progression; ARR_2_PB-Myc that shows high levels of Myc, with a PIN progression and then invasive carcinoma, by the third month of age [45]; ARR_2_PB-FGFR1 models that show the physiological role of Fibroblast growth factor receptors 1 and 2 (FGFR1-2) that become upregulated in 40% of poorly differentiated PCa [46]; PB-Ras [47], PB-Neu [48], and ARR_2_PB-ERG [49] instead were generated inducing an overexpression of any oncogenes that have an important role in different human tumor disease, as Neu in breast cancer [63] or Ras in any other [64].

*Knockout models* (Table 3) have been carried out by a different strategy that led to induce silencing of any tumor suppressor genes through deletion of whole sequences or part of them, essential for their activities. In 1993 RAR*γ*, the first KO mouse model of prostate cancer, was born; it was not able to develop PCa but only squamous metaplasia in mouse prostate and seminal vesicles [50]. Kip1 knockout mouse instead was generated through the loss of p27 [51]; this model showed hyperplasia of some organs among which is prostate. For these reasons it represents a very good model of BPH (benign prostatic hyperplasia). In 1999 Nkx3.1 KO model [52] that showed several prostatic phenotype as hyperplasia and dysplasia was generated but no tumor lesions have been detected. Finally we report the Pten KO, probably the best model to study PIN progression [53], without metastatic proliferation. The Pten-null background has been intercrossed with other KO to generate a more confident mouse model of PCa, for instance, Pten-null × p27 [54] that showed the prostatic adenocarcinoma features or Pten-null × p53 that instead displays HGPIN [55]. Different conditional knockout models of PCa have been developed, during last years, using the Cre-loxP recombination system to excise specific DNA regions in somatic cells, through the gene regulation by tissue specific promoter as Probasin or PSA. Also these models are generated by specific gene silencing of PTEN [56], pRB [57], p53 [58], Apc [59], IGF-1 [60], and Brca2 [61].

## 3. Prostate Tumor PDX Mouse Models

Preclinical prostate cancer mouse models described so far fail to reproduce with high fidelity the different stages of tumorigenesis and the progression of disease observed in the clinic. Normally the xenograft cell lines lose the original tissue architecture of the site of origin, with related impaired physical and biochemical pattern of interaction with the environment, different gene expression, and altered response to pharmacological treatments [65, 66]. Because of the issues described so far, PDX seems to be a good preclinical model to reproduce tumor features, maintaining more similarities; in this way many studies have shown the quality of PDX models highlighting histological compatibilities [67, 68], saved tumor cells heterogeneity [69], similar gene expression [70], gene variants [71], and miRNA pattern [72]. On the contrary there are no other limitations that suggest why these models are not still widely used, such as the more difficult tissue implantation procedures, especially in orthotopic mouse models [73, 74]; the site of implantation choice, in order to provide a good vascularization and cytokines/growth factors supply [73]; an average graft latency period from two to twelve months [75]; an average value of 23–75% of engraftment rates, strictly dependent on tumor aggressiveness [70]. We found studies that demonstrate a strong correlation between poor diagnosis and engraftment rates [68]. More authors suggest making some different serial engraftments (<10) to increase the rates of mouse models, preserving the genetic integrity of parental tumor [76].

The human prostatic tumor xenotransplantation has been firstly described by Gittes in 1980 that reported high take rates (TR = 50%) with the maintenance of many histological human tumor features in athymic nude mice [77]. Subsequently other studies showed a discouraging TR = 0–2% obtained using moderate/well-differentiated carcinomas [78] that poorly increase transplanting pelvic lymph node metastatic tumors supplemented with T-pellets (TR = 10%) [23]; all of the experiments described so far have carried out choosing, as a preferred strategy, the subcutaneous way of implantation, on the mice flank. Reid et al. have increased the TR in their studies, until hundred percent, treating mice with sheep or rabbit anti-mouse interferon serum (globulin fraction) [79]. These latter results have shown that the use of athymic nude mice did not improve the procedure and so the authors decided to change the immunodeficient mice strain, now choosing the NMRI or CB17-NOD athymic nude mice which have a reduced NK activity; Wang et al. [80] described a TR = 58,1% using these strains also supplemented with T-pellet, but in this work the authors decide to investigate also the other way of implantation: subrenal capsule (TR = 93,4%) and orthotopical (TR = 71,9%).

From a histological point of view Gittes [77] showed for the first time a study that demonstrated the development of squamous metaplasia and interstitial fibrosis in the PCa SC xenograft mouse models. They underlined any different modifications in the insert, from the parental carcinoma. Subsequently, for many different tumors, the high cytohistopathological similarity before and after the transplantation has been demonstrated [79, 81, 82]; in addition, both of them had the same Gleason score [83]. Moreover other studies also reported a substantial matching for the PAP and PSA expression in serum, as biomarkers [84, 85]. In most of this work also the metastatic pattern of the different models has been studied in depth; for instance, Hoehn et al. [84] did not find metastasis in any organs during necropsy in PC-82 tumor heterotransplant. On the other hand Lubaroff et al. [86] reported lung metastasis in SCID mice.

According to all of the reported studies so far, it can be affirmed that currently the subrenal capsule (SRC) represents the best way of transplantation because of the high TR; this site represents the gold standard thanks to a high vascularization, a good interstitial fluid pressure, and a high lymphatic flow; those ensure an even nutrients, hormones, oxygen, and growth factors supply [87]; however this site permits the easy placement of an insert with right size [88]. Lin et al. described the SRC grafting procedure using small cutting tumor pieces (1 × 3 × 3 mm^3^ in size) implanted in male NOD/SCID and supplemented with testosterone. After 3 to 6 months of growth the animals were sacrificed and the masses were harvested and reimplanted in SRC. The xenograft pieces were maintained for up to 3 years, increasing the aggressiveness during each passage. Subsequently, in the last passage, the hosts were sacrificed and examined for metastasis in lymph nodes, lungs, liver, kidneys, spleen, and femur [89].

This procedure allows preserving the tumor cells heterogeneity; in fact different studies, focused on prostatic cancer but also on many other different tumors, have shown that during the first phases of xenotransplantation the various tumor cells subpopulation are subjected to an anoxia status caused by the total absence of vascularization that make a selective pressure on them and only the most resistant cells variant can tolerate this environment; the SRC implantation attenuates these hard conditions. This statements have been supported by different studies that confirmed the high similarity between SRC xenograft and the parent tumors in terms of androgen sensitivity, histopathology, biomarkers expression, and metastatic potential [90–93]. Furthermore it has been demonstrated that also the subcutaneous reengraftment, in NSG or NOD-SCID mice, is improved after a well SRC establishment of the insert (TR ~ 95%); this procedure is used to better control the tumor growth, therapeutic responsiveness, or metastatic potential [94].

Given this data we can consider the PDX mouse models like a powerful tool to study the biology of cancer from early stages of tumor progression, until the metastatic dissemination, without losing architectural tissue features or molecular and genetic basis of the disease.

## 4. The “Big Things” in PCa Modelling: PDX 3D Spheroids and Humanized Mice

To date the research on prostate cancer has been hindered not only by the lack of relevant tumor models for clinical use, but also by the heterogeneity of cancer patients. During the last years many studies have investigated the use of PDX derived cell lines to generate disease models; in particular the use of 3D cellular culture was favored with respect to using the 2D monolayer cell lines. 3D spheroids more closely resemble in vivo tissue in terms of cellular communication and the development of extracellular matrices, while standard 2D cell cultures are inadequate representations of this environment, which often makes them unreliable predictors of in vivo drug efficacy and toxicity [18, 95–97]. These evidences reveal that the cell stimulation by environment represents a crucial feature to generate a better disease model; for instance, the bone metastatic PCa cells, whether derived from cell lines or primary PDX tumor tissue, show poor viability and the inability to grow in 2D culture; this is an established phenomenon that indicates the lack of critical components from the bone microenvironment upon which these highly adapted cells depend [98, 99]. To overcome these critical issues, the PDX cancer cells were encapsulated within three-dimensional hyaluronic acid-based hydrogels (HA); such a system has demonstrated that the hydrogel maintains the viability of the cells with the native PDX continuous expression of the androgen receptor [17]. The hydrogel encapsulation provides the means to fully recapitulate the tumor microenvironment with precise, tunable control over architectural and mechanical cues and/or critical cell−extracellular matrix interactions, specifically, as a ubiquitous component of the bone marrow where bone metastatic PCa cells reside. HA plays an active role in regulating several biological processes, including tumorigenesis, strongly justifying its use as an extracellular matrix analogue for culturing bone metastatic tumor cells in vitro [100]. Fong et al. showed that 3D PDX PCa cells exhibited an increased resistance to docetaxel as compared to a standard cell line commonly used in PCa research. The discoveries translation from mouse models to clinical trials has been hampered by the genetic differences between human and inbreed mouse strains and also by the inability to recapitulate human immunological system; for these reasons new and more performing humanized models are required; these tools represent useful ground-breaking platforms for cancer research. To generate humanized mice with functional human immune system, animals with severe immunodeficiency as NOD scid gamma (NSG*™*) are used; they have a severe combined immune deficiency mutation (scid) and IL2 receptor gamma chain deficiency, resulting in a lack of mature T cells and B cells and impaired NK cells, and are deficient in cytokine signalling [101, 102]. In the last few years, in order to eliminate the remaining adaptive and innate immunity in host mice, the animals are inoculated with Human Peripheral Blood Mononuclear Cells (PBMCs) or hematopoietic stem cells CD34+ (HSCs) to create a stable human immune system. PDX-engrafted humanized-NSG models represent the next step in cancer modelling, because they offer a unique platform to examine human T cell-dependent and B cell-dependent responses against clinically relevant tumors and to induce the immune system action versus specific tumor types. In PCa research these models can be considered as a highly validated instrument in human tumor microenvironment studies, also to test tumor response to both Standard Oncologic Cares treatments and novel compounds. They represent an affordable approach to validate single immune-based as well as preclinical combination therapies and allowing new individualized cancer treatments [102, 103].

## 5. Conclusion

Major limitation in prostate cancer research is the lack of relevant preclinical models, which allow studying the molecular mechanisms of tumorigenesis. In fact, advanced in vitro and in vivo models represent an indispensable requirement for the development of new therapeutic strategies. Currently there is a continued research for innovative PCa in vivo models with greater fidelity to disease, which try to foster the translation of preclinical findings into the clinic, particularly to satisfy the need to identify new treatments that will eradicate PCa metastases growing in bone.

The xenograft models represent a great tool to study the mechanisms underlying many human tumors, including efficacy of specific treatments, or cancer stem cell-like properties.

Nevertheless they show some limits related to a compromised immune system of the host that instead becomes crucial in human PCa, especially in metastatic dissemination pattern. In addition, using stabilized or primary tumor cell line, in xenograft, the complete bypassing of all the tumor initiation stages as the interaction with microenvironment or angiogenesis process has been observed. Using cell lines derived from human metastasis, also the metastatic development was impaired [104].

The allograft mouse models results are widely used because they overcome the limits represented by the use of an immunosuppressed host; in this way it is possible to study in depth the interaction between tumor progression and immune system; on the other hand they do not show any limitations linked with the use of not human biological materials.

The GEMMs have been allowed to reproduce the similar genetic alterations that occur in human. So we can divide all the transgenic mouse models discussed in two different categories, on the basis of typical features. The first-generation GEMMs showed an aggressive phenotype, often with metastatic proliferation, castration-resistant PCa, due to the SV40 Tag action on p53 and pRB (not present in human), and a high incidence of neuroendocrine aberrant transformation. Instead the second generation, created through ectopic expression of endogenous oncogenic effectors, seems to not be able to reproduce the different human disease stages of PCa in mouse.

The last generation of preclinical mouse models is represented by PDXs that offer a powerful means for studying biological pathways in cancer and for testing new drugs. Given the data reported, they seem to closely reproduce the progression of PCa, representing a better predictive model compared with all those that are generated by established or primary cell line transplantation, further tracking the various passages of tumor progression, from implantation to metastatic dissemination. Instead, especially in PCa models, we have described the different issues that occur during the procedures, correlated with the choice of the site of implantation; this leads to a less broad availability of prostatic tumor PDX models than others, as breast cancer PDX, that can be developed through transplantation in different sites: interscapular or mammary fat pad and renal capsule.

To date many scientists have developed different tools to overcome the limits of the PDXs; one of the most promising ones seems to be the in vitro PDX 3D cell cultures. This procedure considers all the PDX passages, with the advantage of reducing the number of animal hosts [17], subsequently transferring all the experimental conditions in 3D cell cultures. This technology represents a valid preclinical model that bypasses the issues of 2D monolayer cell cultures; it may be used for a rapid and high throughput platform to assess drug efficacy and to find new predictive biomarkers for novel targeted therapies [91].

The models discussed here have considerable importance to understand the tumor pathogenesis and the complex biology of PCa. They also represent a promising support to enhance development of new approaches to prevention, detection, and treatment of this malignancy. These tools will be useful for a better and faster pharmacological screening in drug discovery and personalized medicine. Furthermore, PDX mouse models will allow a more profound knowledge about a still unexplored area of research in preclinical mouse models generation such as the interaction between human xenogenic stroma and the neoplasm. In the near future, new models for translational research are expected to be generated; they will aim to ameliorate the correlation between results obtained in animal models and human patients. Given the data it could be necessary to generate new preclinical mouse models to enhance understanding of PCa development and progression to metastasis.

## Figures and Tables

**Table 1:** Prostatic mouse models of engraftment.

Type	Background strain	Year	Features	Disadvantages	Reference
Xenograft	Nude, SCID, NOD-SCID, NOG/NSG, RAG	1996–2006	High take rate, low costs, LNCaP, LNCaP-LN3, PC-3, PC-3M cell lines and human prostatic tissues implanted	Immune-compromised mice tested	[23–27]

Allograft	C57BL/6	1997–1994	High take rate, low costs, use of immune-competent mice; TRAMP-C1/2/3, PTEN-CaP8 cell lines implanted	Low translational potential	[31, 32, 38]

Allograft	BALBC/c	2004	High take rate, low costs, use of immune-competent mice; use of PNEC30 cell lines	Low translational potential Neuroendocrine originated cells	[34]

**Table 2:** Genetically engineered mouse models (GEMM) of prostate cancer.

Name	Background strain	Year	Features	Disadvantages	Reference
C3(1)-Tag	FVB/N	1994	PHH (3 mths) PCa (7–11 mths)	Sporadic metastases, aspecific breast cancer in female (12 wks)	[37]

TRAMP	C57BL/6	1994	PHH (8 wks), PIN (18 wks) Lymph. metastases (28 wks)	Neuroendocrine originated tumors	[38]

FG-Tag	C57BL/6	1996	Accurate model of castration-resistant PCa	Aspecific adrenocortical tumors in 50% of female mice	[39]

LPB-Tag (LADY)	CD-1	1997	Accurate model for all stages of PCa studies	Neuroendocrine originated foci in liver, lymph., and lung metastases	[40]

LPB-Tag/ARR_2_PB hepsin	C57BL/6JxCBA	2004	Increased migratory ability	Neuroendocrine originated cells forming liver metastases	[41]

Mt-PRL	C57BL/6JxCBA-f2	1997	Appropriate model to study BPH	Rare PCa progression, no metastases	[42]

BK5-IGF1	FVB × ICR	2000	PIN (6 mths), PCa (9 mths)	Not metastases, off-target effects	[43]

PB-mAR	FVB	2001	Microinvasive HGPIN, useful in PCa studies about androgens	Rare metastases	[44]

ARR_2_PB-Myc	FVB	1999	From PIN to PCa in 2 mths Regression after castration (8 mths)	Not metastases observed	[45]

ARR_2_PB-FGFR1	FVB	2003	Hyperproliferation and PIN	Failed in the later stages of disease	[46]

PB-Ras	FVB/N	2004	Did not progress further than PIN	Intestinal metaplasia Thickened fibromuscular stroma	[47]

PB-Neu	C57BL/6JxCBA	2006	Similar to human acinar type	Low translational potential (rare cases of Neu alteration in human PCa)	[48]

ARR_2_PB-ERG	FVB	2008	PIN (12–14 wks)	Take rate of 38%	[49]

PHH: prostatic epithelial hyperplasia; PIN: prostatic intraepithelial neoplasia; BPH: benign prostatic hyperplasia.

**Table 3:** Prostatic knockout and conditional knockout mouse models.

Name	Type	Background strain	Year	Features	Disadvantages	Reference
RAR*γ*	KO	C57Bl6F1	1993	Squamous cell metaplasia in prostate and seminal vesicles	PCa not developed; mucosal alteration, inflammation	[50]

Kip1	KO	C57BL/6	1996	Hyperplasia of prostate acinar cells (similar to that seen in PPH)	Hyperplasia of multiple organs; not metastases	[51]

Nkx3.1	KO	129/SvImJ C57Bl/6J	1999	Severe hyperplasia/dysplasia with haploinsufficiency (in heterozygous)	Altered morphogenesis of salivary glands	[52]

PTEN	KO	C57BL6/J	1998	PIN development	Off-target alteration: gonads, lymphoid cells, uterus, thyroid, adrenal glands	[53]

PTEN × p27	KO	C57BL6/J	2001	PCa progression with high take rate	Off-target alteration: endometrium, thyroid, adrenal glands	[54]

PTEN × p53	KO	129 SvJ	2009	HGPIN	Off-target alteration: gonads, lymphoid cells, uterus, thyroid, adrenal glands	[55]

PSA^Cre^Pten^flox^	Conditional KO	FVB	2005	Various stages of hyper/displasia High translational potential	Rare lymph nodes metastases	[56]

PB^Cre^Rb^flox^	Conditional KO	C57BL/6	2004	Hyperplasia with loss of basement membrane integrity	Insufficient transformation	[57]

PB^Cre4^p53^flox^Rb^flox^	Conditional KO	C57BL/6xDBA2 × FVB/N	2006	Metastatic carcinoma (also with distant metastases)	Rare skeletal metastases	[58]

PB^Cre4^Apc^flox^	Conditional KO	C57Bl/6J and 129Sv/J	2007	PIN followed by locally adenocarcinoma	Not distant metastases displayed	[59]

PB^Cre4^IGF-1^flox^	Conditional KO	FVB/NJ	2008	Hyperplasia	Insufficient transformation	[60]

PB^Cre4^Brca2^flox^p53^flox^	Conditional KO	Mixed genetic background	2010	Early HGPIN (from 12 mths) due to high DNA damage	Rare metastasis	[61]

HGPIN: high-grade prostatic intraepithelial neoplasia.
